# Quantitative Impact of Traditional Open Surgery and Minimally Invasive Surgery on Patients’ First-Night Sleep Status in the Intensive Care Unit: Prospective Cohort Study

**DOI:** 10.2196/56777

**Published:** 2024-11-22

**Authors:** Chen Shang, Ya Yang, Chengcheng He, Junqi Feng, Yan Li, Meimei Tian, Zhanqi Zhao, Yuan Gao, Zhe Li

**Affiliations:** 1 Department of Critical Care Medicine Renji Hospital School of Medicine, Shanghai Jiao Tong University Shanghai China; 2 Department of Infection Control Renji Hospital School of Medicine, Shanghai Jiao Tong University Shanghai China; 3 School of Biomedical Engineering Guangzhou Medical University Guangzhou China; 4 Department of Critical Care Medicine Peking Union Medical College Hospital Beijing China; 5 Institute of Technical Medicine Furtwangen University Villingen-Schwenningen Germany

**Keywords:** sleep quality, wearable sleep monitoring wristband, intensive care unit, minimally invasive surgery, traditional open surgery

## Abstract

**Background:**

The sleep status of patients in the surgical intensive care unit (ICU) significantly impacts their recoveries. However, the effects of surgical procedures on sleep are rarely studied.

**Objective:**

This study aimed to investigate quantitatively the impact of traditional open surgery (TOS) versus minimally invasive surgery (MIS) on patients’ first-night sleep status in a surgical ICU.

**Methods:**

Patients transferred to the ICU after surgery were prospectively screened. The sleep status on the night of surgery was assessed by the patient- and nurse-completed Richards-Campbell Sleep Questionnaire (RCSQ) and Huawei wearable sleep monitoring wristband. Surgical types and sleep parameters were analyzed.

**Results:**

A total of 61 patients were enrolled. Compared to patients in the TOS group, patients in the MIS group had a higher nurse-RCSQ score (mean 60.9, SD 16.9 vs mean 51.2, SD 17.3; *P*=.03), self-RCSQ score (mean 58.6, SD 16.2 vs mean 49.5, SD 14.8; *P*=.03), and Huawei sleep score (mean 77.9, SD 4.5 vs mean 68.6, SD 11.1; *P*<.001). Quantitative sleep analysis of Huawei wearable data showed a longer total sleep period (mean 503.0, SD 91.4 vs mean 437.9, SD 144.0 min; *P*=.04), longer rapid eye movement sleep period (mean 81.0, 52.1 vs mean 55.8, SD 44.5 min; *P*=.047), and higher deep sleep continuity score (mean 56.4, SD 7.0 vs mean 47.5, SD 12.1; *P*=.001) in the MIS group.

**Conclusions:**

MIS, compared to TOS, contributed to higher sleep quality for patients in the ICU after surgery.

## Introduction

Impaired sleep quality is a common and distressing problem among surgical patients, particularly among those admitted to the intensive care unit (ICU) [1,2]. Sleep disruption can lead to delirium, compromised immune function, and extended wound recovery, ultimately resulting in adverse outcomes [2].

Several surgical factors can impair sleep quality, including the magnitude of the surgical intervention, physical pain, stress related to the operation, and concerns about pathology results [1,3]. For instance, patients who underwent major abdominal surgery may lose up to 80% of their total sleep time during the first few postoperative nights [4]. Minimally invasive surgery (MIS), which involves smaller incisions and fewer complications, has recently become a viable alternative to traditional open surgery (TOS), as recommended by the Enhanced Recovery After Surgery guidelines [5,6]. However, the impact of MIS on sleep status remains poorly understood.

The accurate measurement of sleep in ICU settings is hindered by various technical obstacles. While polysomnography (PSG) is widely regarded as the most reliable method for assessing sleep, its utilization is constrained by several factors. First, PSG requires special equipment, as well as complex operational procedures, which can be challenging to implement in an ICU setting. Additionally, artifacts caused by other monitoring devices and medical interventions can interfere with PSG recordings, making it difficult to obtain accurate sleep data. Furthermore, the interpretation of PSG results requires skilled personnel, which may not always be readily available in an ICU environment [7,8]. Lastly, PSG is often uncomfortable for patients and can even exacerbate sleep disturbances [9]. Consequently, it is not surprising that questionnaires, such as the Richards-Campbell Sleep Questionnaire (RCSQ), are frequently used for sleep assessment [8]. However, the accuracy and reliability of these questionnaires are undermined by a range of factors. Patients in the ICU may have cognitive, communicational, and physical limitations that hinder their ability to accurately respond to the questions. On the other hand, nurses may face difficulties in accurately assessing patients’ sleep-wake states due to their workload or other reasons [3,10]. In practice, there is a pressing clinical need for a convenient and objective technique to enhance the validity of bedside sleep assessment.

The field of sleep monitoring has witnessed rapid advancements in consumer technology, with wearable devices becoming increasingly popular among the general public. Previous studies have demonstrated the promising validity of wearable wristbands in identifying patients’ sleep patterns compared to PSG [11]. Moreover, it is conceivable that sleep monitoring wristbands could serve as potential tools for evaluating sleep in the ICU [12]. Empirical wristbands, equipped with accelerometers (actigraphy), offer a noninvasive and affordable solution for continuous sleep monitoring [13]. However, there is a risk that wristbands may overestimate total sleep time in patients who are sedated [13]. Modern wearable sleep monitoring devices integrate cardiopulmonary coupling technique and analysis software, enabling more accurate identification of sleep stages, including sleep depth, rapid eye movement (REM) sleep, deep sleep, and light sleep [14]. This offers the opportunity to assess postoperative sleep patterns in patients after they wake up from surgery.

This study investigates postoperative ICU patients’ sleep patterns on the surgical night using wearable wristbands and RCSQs to quantify the impact of TOS and MIS procedures on sleep status. The findings will provide an objective basis for tailoring sleep management strategies in the surgical ICU.

## Methods

### Study Population

This prospective cohort study was conducted in an 18-bed surgery ICU at Renji Hospital, Shanghai, China. Patients aged 18 years or older who underwent gastrointestinal, biliary and pancreatic, urology, or orthopedic surgery and were transferred to the ICU from June 2022 to September 2022 were screened. Patients with 1 or more of the following conditions were excluded: neurological or mental disorders, dementia, chronic sleep disorders, failure to achieve postanesthesia resuscitation and to be extubated before 6:00 PM, use of remifentanil exceeding 0.03 μg/kg/min, hemodynamic instability (defined as an irreversible systolic blood pressure below 90 mm Hg), or at a high risk of reoperation during the study period as determined by the attending physician.

### Ethical Considerations

This study was approved by the Ethics Committee of Renji Hospital (KY2022-145-A). All data have been anonymized to protect patient privacy, and participants benefit from long-term follow-up care as compensation. Written informed consent was obtained from patients or their legal representatives.

### Sleep Monitoring

The sleep monitoring period was from 9:00 PM on the night of the surgery to 7:00 AM on the following day. A wearable wristband (HONOR BAND 6, Huawei Technologies Co., Ltd) was used to collect sleep data. At 7:00 AM the next day, the bedside nurse removed the wristband and transferred the encrypted sleep data to a customized application. The nurse-RCSQ was then completed by the bedside nurse, while a nonbedside nurse interviewed the patient and filled out the self-RCSQ.

### Data Collection

Patient demographic data, medication history, operation information, ICU treatments, and sleep parameters were collected. Both patient- and nurse-reported RCSQ scores were rated from 0 (Bad) to 100 (Good) on a visual analog scale [15] (Multimedia Appendix 1). Sleep data from wearable wristbands were automatically translated into sleep parameters, including Huawei sleep score, light sleep, deep sleep, REM, and wakefulness, with customized, built-in software that translates cardiopulmonary coupling and accelerometry data into sleep-wake periods [14].

### Study Group

Patients were divided into two study groups based on the surgical approach: (1) MIS group—patients who underwent laparoscopic or robotic-assisted techniques via small skin incisions [5] and (2) TOS group—patients who underwent traditional, open surgical techniques.

### Outcome

The primary outcome was the sleep scores of patients who underwent MIS or TOS. The secondary outcomes included the consistency of sleep status assessed by wearable wristbands and RCSQs, as well as quantitative sleep parameters from the wristbands.

### Statistical Analysis

Based on preliminary data, and based on a .05 α level and 80% power, we calculated a sample size of 25 per group to observe an average increase of 10 points in Huawei and RCSQ sleep scores in patients who underwent MIS compared to patients who underwent TOS [16]. All values are presented as mean (SD), median (IQR), number, and percentage (%) as appropriate. Continuous data were analyzed using 2-tailed *t* tests or the Mann-Whitney *U* test, as appropriate. Bland-Altman concordance analysis was performed to assess the agreement between the 2 assessments. Multivariable linear regression was conducted to estimate the effects of surgical mode on sleep-related indexes. A 2-sided *P* value <.05 was considered statistically significant. Anthropometric data and measurements were analyzed using R software (version 4.10; R Foundation for Statistical Computing).

## Results

### Characteristics

A total of 61 patients received sleep assessments; 28 (46%) patients underwent MIS, while 33 (54%) patients underwent TOS. Table 1 presents the demographic and perioperative characteristics between the MIS and TOS groups. The MIS group had a lower proportion of upper abdominal surgeries and a higher proportion of lower abdominal and pelvic surgeries compared to the TOS group.

**Table 1:** Demographic and perioperative characteristics of enrolled patients.

Variables			MIS^a^ (n=28)		TOS^b^ (n=33)
**Age (year), mean (SD)**			65.1 (11.2)		69.7 (8.0)
**Male sex, n (%)**			17 (61)		21 (64)
**APACHE^c^ score, median (IQR)**			15.5 (15.0-18.0)		15 (13.8-16.0)
**Sedation and analgesia^d^, n (%)**			10 (36)		12 (36)
**Drainage tube number, median (IQR)**			7 (6-8)		7 (6-8)
**Medical interventions^e^, median (IQR)**			10.5 (9.0-12.0)		11.0 (10.0-12.0)
**Surgical site, n (%)**					
	Upper abdomen	10 (36)		24 (73)	
	Lower abdomen and pelvis	12 (43)		2 (6)	
	Orthopedic	6 (21)		7 (21)	

^a^MIS: minimally invasive surgery.

^b^TOS: traditional open surgery.

^c^APACHE: Acute Physiology and Chronic Health Evaluation.

^d^Sedation and analgesia: use of dexmedetomidine and remifentanil during the postoperative period.

^e^Medical interventions: noninvasive blood pressure, blood drawing and injection, and other medical procedures.

### Comparison of Postoperative Sleep Scores

**Figure 1:**
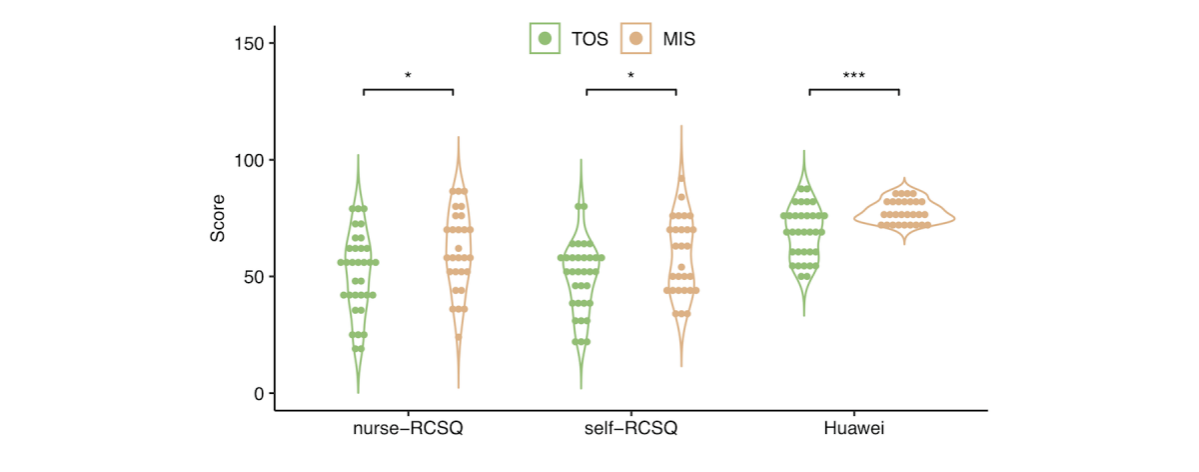
Violin plots displaying the distribution of individual values of the nurse-RCSQ, self-RCSQ, and Huawei sleep scores; differences among groups were assessed by 2-tailed t tests. *P＜.05, ***P＜.001. MIS: minimally invasive surgery; RCSQ: Richards-Campbell Sleep Questionnaire; TOS: traditional open surgery.

**Figure 2:**
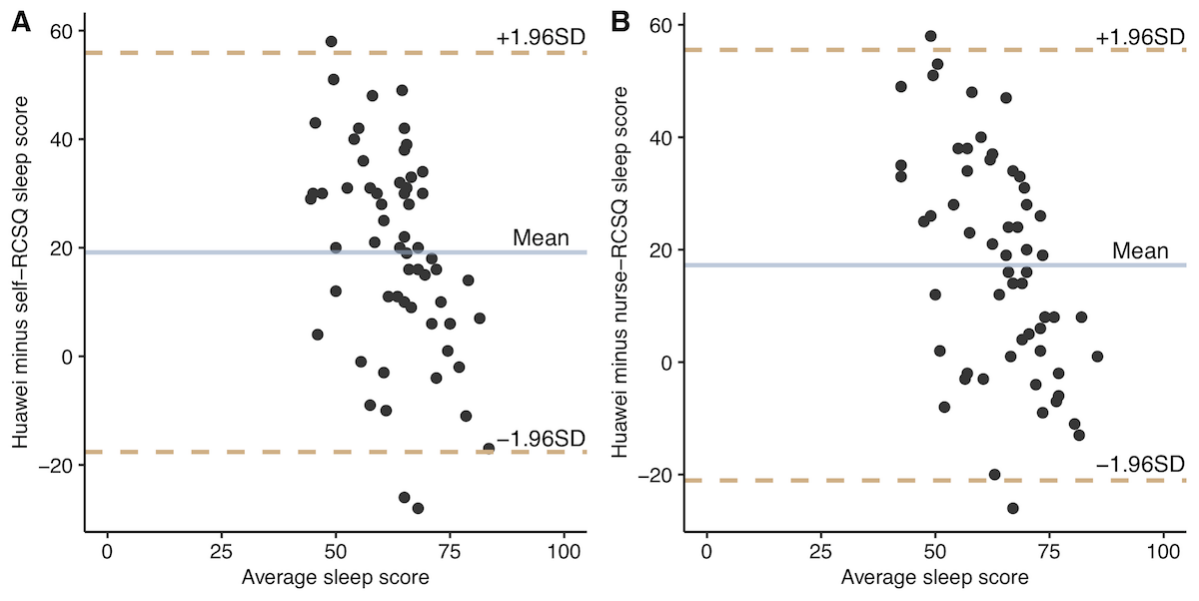
Bland-Altman analysis between Huawei and RCSQ sleep scores. Horizontal line are drawn at the mean difference and at the mean difference plus and minus 1.96 SD of the differences, with the x-axis reporting average levels of sleep scores. (A) Huawei sleep monitoring wristband versus self-RCSQ assessment of total sleep score, with 95% (58/61) of the points within the clinically acceptable consistency boundary. (B) Huawei sleep monitoring wristband versus nurse-RCSQ assessment of total sleep score, with 97% (59/61) of the points within the clinically acceptable consistency boundary. RCSQ: Richards-Campbell Sleep Questionnaire.

An average increase of 10 points was observed in the nurse-RCSQ score (mean 60.9, SD 16.9 vs mean 51.2, SD 17.3; *P*=.03), self-RCSQ score (mean 58.6, SD 16.2 vs mean 49.5, SD 14.8; *P*=.03), and Huawei sleep score (mean 77.9, SD 4.5 vs mean 68.6, SD 11.1; *P*<.001) in patients who underwent MIS compared to patients who underwent TOS (Figure 1). After adjusting for baseline information including the surgical site, the effect of surgical mode (MIS or TOS) was significant for the self-RCSQ score (*P*=.03) and Huawei sleep score (*P*<.001), as shown in Table 2. Furthermore, the Bland-Altman analysis demonstrated a high level of consistency, with over 95% agreement between the Huawei sleep score and self-RCSQ (58/61, 95%) and nurse-RCSQ scores (59/61, 97%; Figure 2).

**Table 2:** Multivariate linear regression analysis of surgical mode on nurse-RCSQ^a^, self-RCSQ, and Huawei sleep scores.

Score	Adjusted β^b^	SE	*t* statistic (*df*)	*P* value
Nurse-RCSQ score	10.012	5.416	1.849 (50)	.07
Self-RCSQ score	11.382	4.929	2.309 (50)	.03
Huawei sleep score	10.970	2.740	4.003 (50)	<.001

^a^RCSQ: Richards-Campbell Sleep Questionnaire

^b^Models were adjusted for age, sex, Acute Physiology and Chronic Health Evaluation (APACHE) score, use of sedation and analgesia, number of drainage tubes and medical interventions, and surgical site.

### Quantitative Impact of Surgical Mode on Sleep Status

Quantitative analysis on the duration and depth of sleep using Huawei software is presented in Table 3. The MIS group had a 1-hour longer total sleep time (mean 503.0, SD 91.4 vs mean 437.9, SD 144.0 min; *P*=.04) and a 25-minute longer REM sleep time (mean 81.0, SD 52.1 vs mean 55.8, SD 44.5 min; *P*=.047) compared to the TOS group. Similarly, patients who underwent MIS had a higher deep sleep continuity score (mean 56.4, SD 7.0 vs mean 47.5, SD 12.1; *P*=.001) compared to patients who underwent TOS. However, the deep sleep time, light sleep time, deep sleep ratio, light sleep ratio, REM sleep ratio, and awakening frequency showed no significant differences in patients who underwent MIS versus TOS(all *P*>.05). Multiple linear regression showed that the effects of the surgical mode (MIS or TOS) on Huawei sleep score, total sleep time (*P*=.03), REM sleep time (*P*=.02), and deep sleep continuity score (*P*=.02) were all significant after adjustment for age, sex, Acute Physiology and Chronic Health Evaluation (APACHE) score, use of sedation and analgesia, number of drainage tubes and medical interventions, and surgical site (Table 4).

**Table 3:** Quantitative sleep analysis of Huawei wearable data.

Variables	MIS^a^ (n=28), mean (SD)	TOS^b^ (n=33), mean (SD)	*P* value^c^
Total sleep time (min)	503.0 (91.4)	437.9 (144.0)	.04
Deep sleep time (min)	145.7 (55.6)	125.6 (79.7)	.25
Light sleep time (min)	282.0 (92.3)	255.1 (94.5)	.27
REM^d^ sleep time (min)	81.0 (52.1)	55.8 (44.5)	.047
Deep sleep ratio (%)	29.7 (7.8)	27.2 (12.4)	.33
Light sleep ratio (%)	55.8 (11.2)	61.1 (17.0)	.15
REM sleep ratio (%)	14.5 (6.9)	11.7 (7.3)	.13
Deep sleep continuity score	56.4 (7.0)	47.5 (12.1)	.001
Awakening frequency	2.4 (2.0)	2.5 (2.3)	.87

^a^MIS: Minimally invasive surgery.

^b^TOS: Traditional open surgery.

^c^Differences among groups were assessed using 2-tailed *t* tests.

^d^REM: rapid eye movement

**Table 4:** Multivariate linear regression analysis of surgical mode on Huawei sleep indexes.

Variables	Adjusted β^a^	SE	*t* statistic (*df*)	*P* value
Total sleep time	83.314	37.356	2.230 (50)	.03
REM^b^ sleep time	36.900	15.501	2.380 (50)	.02
Deep sleep continuity score	8.126	3.363	2.417 (50)	.02

^a^Models were adjusted for age, sex, Acute Physiology and Chronic Health Evaluation (APACHE) score, use of sedation and analgesia, number of drainage tubes and medical interventions, and surgical site.

^b^REM: rapid eye movement.

## Discussion

### Principal Findings

This study reveals that patients who underwent MIS exhibit better sleep quality, characterized by longer total sleep time, increased REM sleep duration, and higher deep sleep continuity compared to those who underwent TOS. Furthermore, wearable sleep monitoring wristbands provided quantitative sleep data that align with RCSQ assessments. These findings provide initial support for the use of modern sleep monitoring technology in managing sleep in patients in surgical ICUs.

Disruptions to regular sleep-wake cycles can lead to a range of complications, including anxiety, immune disorders, prolonged hospitalization, and social reintegration difficulties [17]. MIS techniques reduced invasiveness and promoted faster recovery, even in critically ill patients. Our results show a significant 10-point improvement in sleep scores for patients who underwent MIS compared to TOS, consistent with previous findings indicating that major surgeries can exacerbate sleep disturbances compared to minor ones [18]. Our findings can be attributed to a combination of physical and psychological factors. Laparoscopic repair is associated with reduced postoperative pain and faster recovery while minimizing psychological stress, as shown by lower anxiety and stress scores [19,20]. Consequently, patients who underwent MIS require fewer narcotics, including opioids, on postoperative days 0 and 1, which reduces sleep disturbances caused by anesthesia use [18,21]. At the molecular level, MIS minimizes mechanically induced damages and immune responses, leading to reduced systemic release of acute phase proteins, leukocytosis, and interleukin-6 [22]. This reduction in inflammatory responses may affect circadian rhythm and sleep quality by impacting vagal projections, sympathetic ganglia, and the blood-brain barrier [23,24]. Our local quality control report in 2021 also found that patients who underwent MIS had higher scores for sleep and overall in-hospital satisfaction compared to patients who underwent TOS.

Our Huawei data show that patients who underwent MIS experienced a significant reduction in sleep disruption, with an additional hour of total sleep time, an extra 25 minutes of REM sleep, and improved deep sleep continuity. REM sleep is a critical component of sleep quality, characterized by active brain activity, vivid dreaming, and relaxed skeletal muscles [25]. Major surgeries often disrupt REM sleep, with reports of significant suppression following open heart surgery [26] and a decline of REM sleep from 18% to 0% after open cholecystectomy on the night of the surgery [27]. Sleep continuity is a key indicator of sleep quality, as disruptions can lead to fragmented sleep, impaired memory consolidation, and cognitive dysfunction [28]. In our study, both groups had relatively low sleep continuity, consistent with previous research showing that even sufficient sleep duration can be fragmented in postoperative patients in the ICU [29]. Assessing sleep continuity may be a clinically relevant and reproducible method for evaluating sleep disruption in ICU settings [30].

Quantifying the sleep status of patients in the ICU is challenging due to the difficulties in implementing PSG measurement. Laboratory studies have shown that wristbands exhibit high sensitivity (0.965) and accuracy (0.863) compared to PSG [31]. In critically ill patients, wristbands have been validated in correlating with PSG to identify total sleep time and wakefulness [12,32]. However, patient selection in previous studies had affected monitoring accuracy, as wristbands monitoring has much less specificity for patients who are ventilated compared with those who are not (51% vs 83.7%), possibly due to sedation and constraints [33]. Our study focuses on patients who have regained consciousness after anesthesia and found that Huawei wristband sleep scores show high consistency (>95%) with both self-RCSQ and nurse-RCSQ scores. Our results suggest that this technology is suitable for monitoring sleep patterns in patients after they wake up in the ICU.

Wearable sleep monitoring devices offer a distinct advantage in clinical and research settings for patients in the ICU as they provide both objective, quantitative information on sleep states and continuous monitoring capabilities for future analysis. For instance, these devices can facilitate the investigation of sleep disorders and their correlation with adverse outcomes, such as delirium [13,34]. They offer a valuable early warning system for sleep-related complications and serve as a tool to assess the impact of ICU interventions and medications on sleep quality and patient outcomes. Additionally, long-term sleep monitoring, spanning over several days, and its variability (night-to-night variability) can have profound implications on patient outcomes and the well-being of health care associates [35]. The collaboration between medical and engineering professionals enables the utilization of extensive clinical data to guide the development of hardware and software for these devices, evolving them from wearable health monitors into professional medical devices.

This study, conducted at a single center, has several limitations. First, our sleep monitoring period (between 9 PM and 7 AM) was limited by local sleep and clinical work habits, which may introduce bias. In future research, it would be desirable to conduct continuous sleep monitoring in the ICU to examine sleep patterns over a longer period. Second, the higher absolute value of the Huawei sleep score in our study suggests that if we aim for large-scale clinical use, a validated wearable device scoring system with clear and simple standards is necessary. Finally, a larger, multicenter study is necessary to validate the findings through continuous recruitment.

### Conclusion

Compared to TOS, MIS contributed to a higher sleep quality for patients in the ICU after surgery, manifested as longer sleep time, longer REM sleep time, and better continuity of deep sleep. Wearable monitoring wristbands hold the potential for quantified sleep assessment in ICU settings.

## Appendix

Multimedia Appendix 1 Richards-Campbell Sleep Questionnaire.

## Abbreviations

ICU: intensive care unit
MIS: minimally invasive surgery
PSG: polysomnography
RCSQ: Richards-Campbell Sleep Questionnaire
REM: rapid eye movement
TOS: traditional open surgery

## Data Availability

The data in this study are available from the corresponding author on reasonable request.
