# Supplementary material for: Quantitative Impact of Traditional Open Surgery and Minimally Invasive Surgery on Patients’ First-Night Sleep Status in the Intensive Care Unit: Prospective Cohort Study
Source: J Med Internet Res. 2024 Nov 22;26:e56777. doi: 10.2196/56777 (PMC11624462; doi:10.2196/56777)
Supplement: Multimedia Appendix 1 [file jmir_v26i1e56777_app1.docx]

**Multimedia Appendix 1.** Richards-Campbell Sleep Questionnaire.

| *Measure* | *Question* |
| --- | --- |
| 1. Sleep depth | My sleep last night was: light sleep (0) ... deep sleep (100) |
| 2. Sleep latency | Last night, the first time I got to sleep, I: just never could fall asleep (0) ... fell asleep almost immediately (100) |
| 3. Awakenings | Last night, I was: awake all night long (0) ... awake very little (100) |
| 4. Returning to sleep | Last night, when I woke up or was awakened, I: couldn't get back to sleep (0) ... got back to sleep immediately (100) |
| 5. Sleep quality | I would describe my sleep last night as: a bad night's sleep (0) ... a good night's sleep (100) |

Each question is scored by using a 100-mm visual analog scale in which a higher score is better.
